# SARS Coronaviruses and Highly Pathogenic Influenza Viruses: Safety and Occupational Health for Laboratory Workers

**DOI:** 10.3201/eid1104.041304

**Published:** 2005-04

**Authors:** Jill Taylor, David E. Wentworth, Kristen A. Bernard, Paul S. Masters, Charles V. Trimarchi

**Affiliations:** * New York State Department of Health, Albany, New York, USA

**Keywords:** SARS-CoV, highly pathogenic influenza virus, medical monitoring, laboratory safety, occupational health

**SARS Coronaviruses and Highly Pathogenic Influenza Viruses: Safety and Occupational Health for Laboratory Workers, a workshop**

Wadsworth Center, Albany, New York, USA

September 30, 2004

On July 5, 2003, the World Health Organization (WHO) declared that the global epidemic of severe acute respiratory syndrome (SARS) had been contained. Although several individual natural recurrences of SARS occurred in the respiratory disease season of 2003, these were quickly contained with no person-to-person transmission. Subsequently, during the spring and summer of 2004, 3 episodes of laboratory-acquired SARS occurred, the last of which led to 1 death and raised the specter of extended community spread. These occurrences resulted in considerable discussion regarding the limitation of research on infectious SARS-associated coronavirus (SARS-CoV) around the world and how best to avoid reemergence of the virus from laboratories. Although no analogous laboratory incidents with highly pathogenic strains of influenza virus (HPIV) have been documented, similar concerns exist for this agent.

The Wadsworth Center, the laboratory arm of the New York State Department of Health, supports research laboratories working with SARS-CoV and influenza virus. An occupational health and medical monitoring protocol has been developed to manage potential laboratory exposures and to ensure early detection and containment of an actual laboratory-acquired infection. The protocol addresses medically monitoring workers after a documented laboratory accident, as well as monitoring workers who suffer respiratory infections, even if no laboratory accident has occurred. While we anticipate that the number of protocol breaches leading to laboratory-acquired infections will be low, illness from a community-acquired infection will develop in a laboratory worker at some point. Because of the immediacy and high-profile nature of these issues, a workshop was held on September 30, 2004, at the Wadsworth Center in Albany, New York, to discuss how to make such a protocol practicable in a research setting and to gain input from the scientific community. The workshop attracted 75 participants, with representatives from the Centers for Disease Control and Prevention (CDC) and WHO, as well as scientists from Toronto, Hong Kong, and laboratories across the continental United States. Also in attendance were biosafety and occupational health professionals and representatives from the medical, legal, and insurance professions. All participants had been provided with a copy of the Wadsworth Center draft protocol before the meeting.

Because the audience came from diverse backgrounds, the program began by providing a review of those aspects of SARS-CoV and HPIV biology that attach some risk to working with these agents. Concern is raised by the fact that the viruses are not currently circulating among humans but are highly transmissible once a release occurs. Additionally, SARS-CoV appears to be more stable in the environment than are other human coronaviruses. The importance of reverse genetics in discovering virulence determinants of influenza was emphasized, as was the responsibility that this genetic manipulation places on investigators. The role pigs, and potentially chickens, play as "mixing vessels" for reassortment between viruses from wildfowl and humans was highlighted. The role of chickens in the evolution of the H5N1 virus from the prototype 1996 goose isolate to the current 2004 Z/Z+ genotypes was described, as well as recent findings that the Z/Z+ strains are potent inducers of tumor necrosis factor-alpha in primary human macrophages. That the human H3N2 strain is enzootic in pigs in Asia increases the likelihood of a reassortment event. Also reviewed were the diagnostic assays used to detect SARS-CoV infection; the lack of detailed knowledge about the pathogenesis of this disease makes rapid diagnosis difficult. Available data on limiting hospital transmissions were reviewed, and those factors that put healthcare workers at elevated risk of becoming infected were described. It was suggested that persons >60 years of age and those with coexisting conditions should be excluded from caring for SARS patients and handling SARS-CoV. Mild and asymptomatic cases of SARS infection have been reported; this finding may confound results when laboratory workers are monitored for SARS infection. The third edition of the WHO Laboratory Biosafety Manual is now available (http://www.who.int/csr/resources/publications/biosafety/en/Biosafety7.pdf). WHO's role was defined as providing recommendations and guidelines but not regulating. The evolution of biocontainment practices was described, along with factors to consider in determining the appropriate biosafety level for work with these agents. The need for training and incident reporting was emphasized. An overview of the CDC algorithm for medically monitoring patients potentially infected with SARS-CoV was provided, and the importance of early recognition of disease was confirmed. The risk assessment must be based on both clinical findings and laboratory assays, which may be of limited value early in infection. The audience was reminded that each case is unique and that the algorithm should be used as a guide, adapted to the specific details of a given case, rather than as a protocol to be followed absolutely.

Four panels convened to discuss a range of issues. The first panel reviewed the appropriateness of biosafety practices for work with SARS-CoV and HPIV. The general consensus was that fit-tested N95 masks and goggles are sufficient for most work. However, battery-powered air-purifying respirators should be used when animal studies are conducted. Attendees, panelists, and speakers agreed that respiratory infection following laboratory work, either without an incident or with only a minor, "low-risk" incident, did not require isolating the laboratory worker. The panel agreed that the best decontaminants for hard surfaces were quaternary detergents or a freshly prepared bleach solution.

Panel 2 discussed criteria for risk assessment. Given diagnostic assay limitations early in SARS-CoV infection, multiple rounds of rule-out diagnostic testing should be performed, as well as rule-in tests. When monitoring periods are being determined, one must differentiate between SARS-CoV and HPIV, since the 2 agents have differing incubation periods, and antivirals are available for influenza treatment and prevention. Recurring themes that required discussion included the composition of the risk assessment group, the risk level at which the group should be convened, and to whom the ill employee should be reporting symptoms. Participants generally agreed that the institutional biosafety officer should be involved early in the process, and that the employee's supervisor should not be the only person making the risk assessment, even in a low-risk situation.

Panel 3 noted that virus inactivation measures should be thoroughly validated and that published data for inactivation of SARS-CoV are not complete. Extracted RNA, while infectious under specific conditions, was not seen as posing a practical threat of infection. This panel, composed mostly of scientists from academia, expressed concern that some institutions would not have the resources or mechanisms in place to support the risk evaluation process.

Panel 4 reviewed the issues of balancing employee needs and institutional needs, and several themes emerged. Education and openness were viewed as essential in promoting the appropriate response from an ill employee and in dealing with inquiries about the situation. An ill worker may wish to see his or her own physician, as well as the institutionally designated physician, and this choice should be supported. Involvement of the personal physician may also facilitate institutional access to relevant medical information that could enhance care. Education is also important in determining a person's qualifications to work with these agents. Although an institution may advise that vulnerable persons with particular medical conditions should not work in biosafety level 3 laboratories, these persons cannot be excluded.

The workshop participants concluded that the protocol and subsequent discussion would be of value to other laboratories conducting research with these agents. The scientists at the Wadsworth Center have incorporated comments into the draft protocol, which will be posted on the Wadsworth Center's Web site (http://www.wadsworth.org).

